# Field Effect Sensors for Nucleic Acid Detection: Recent Advances and Future Perspectives

**DOI:** 10.3390/s150510380

**Published:** 2015-05-04

**Authors:** Bruno Veigas, Elvira Fortunato, Pedro V. Baptista

**Affiliations:** 1 Nanomedicine@FCT, UCIBIO, Departamento de Ciências da Vida, Faculdade de Ciências e Tecnologia, Universidade Nova de Lisboa, Campus de Caparica, Caparica 2829-516, Portugal; E-Mail: bmrveigas@gmail.com; 2 CENIMAT/I3N, Departamento de Ciência dos Materiais, Faculdade de Ciências e Tecnologia, Universidade Nova de Lisboa, Campus de Caparica, Caparica 2829-516, Portugal; E-Mail: emf@fct.unl.pt

**Keywords:** field effect, TFT, ISFET, EIS, SiNW, DNA, LAMP, qRT-PCR, label free

## Abstract

In the last decade the use of field-effect-based devices has become a basic structural element in a new generation of biosensors that allow label-free DNA analysis. In particular, ion sensitive field effect transistors (FET) are the basis for the development of radical new approaches for the specific detection and characterization of DNA due to FETs’ greater signal-to-noise ratio, fast measurement capabilities, and possibility to be included in portable instrumentation. Reliable molecular characterization of DNA and/or RNA is vital for disease diagnostics and to follow up alterations in gene expression profiles. FET biosensors may become a relevant tool for molecular diagnostics and at point-of-care. The development of these devices and strategies should be carefully designed, as biomolecular recognition and detection events must occur within the Debye length. This limitation is sometimes considered to be fundamental for FET devices and considerable efforts have been made to develop better architectures. Herein we review the use of field effect sensors for nucleic acid detection strategies—from production and functionalization to integration in molecular diagnostics platforms, with special focus on those that have made their way into the diagnostics lab.

## 1. Introduction

Biosensors represent a well-established field attracting high investments in research and industry, where handheld biosensors are today are successfully becoming household devices for health monitoring. A similar development can be ambitioned for nucleic acid diagnostics since DNA/RNA detection applications have growing demand in various fields, such as pathogen identification, drug screening and diagnosis of genetic diseases [1,2,3]. Amongst the plethora of biosensing platforms, field effect devices (FED) for biological detection have surged in recent years. In particular, the field effect transistor (FET) is one of the most exciting approaches in electrical DNA detection and characterization showing several advantages: small dimensions, fast response, integration into arrays and possibility of low-cost mass production. The simultaneous analysis of various DNA/RNA targets in miniaturized analytical systems has allowed for the development of comprehensive assay platforms ‒ lab-on-chip [3,4]. Such an example is the integrated semiconductor device enabling non-optical genome sequencing that has been subsequently spun into a commercially available DNA sequencing equipment—IonTorrent [5,6].

This review will focus on the use of field effect sensors for nucleic acid detection strategies that have been reported thus far, and provide a critical evaluation of current and future developments of these technologies assisting DNA detection, identification and characterization. Table 1 summarizes the discussed FED-based strategies.

## 2. Field Effect Biosensors Architecture

**Figure 1:**
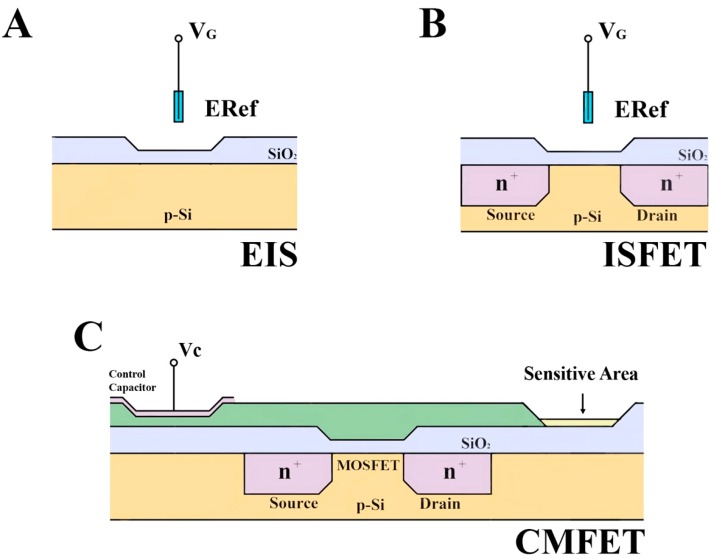
Schematic illustration of thin film device structures: (**A**) EIS—electrolyte-insulator-semiconductor; (**B**) ISFET- ion sensitive field effect transistor; (**C**) CMFET—charge modulated field effect transistor. ERef: reference electrode; Vg: gate voltage; Vc: Capacitor voltage.

Field effect based-sensors are used as the transducer of biorecognition events (DNA/RNA hybridization) mainly following two main designs: (1) a metal-insulator- semiconductor (MIS) capacitor; and (2) metal-oxide-semiconductor field effect transistor (MOSFET) that have been slightly modified over the years to meet the challenges of integrating biological reactions with electronic devices [7,8]. The MIS capacitor is one of the most simple and useful devices in the study of electronic circuits, whose structure is a semiconductor-insulator interface that serves as a model for the development of sensitive layers and/or materials. The electrolyte-insulator-semiconductor (EIS) capacitor is one of these modified designs and has been broadly applied for biosensing. The EIS structure is identical to that of a MIS capacitor but the gate electrode is replaced by an electrolyte and a reference electrode. The insulator, commonly an oxide, is thus directly exposed to the electrolyte so changes in the solution can affect the oxide surface potential and modulate the device’s response (Figure 1A,B).

As described previously for the EIS design, the ion sensitive field effect transistor (ISFET) sensor structure and operation can be related to its electronic counterpart; the MOSFET. Again, replacing the gate electrode with an electrolyte and a reference electrode, the gate dielectric is directly exposed to the electrolyte [9]. This design was initially applied for electrophysiological measurements, allowing detection and quantification of ions, H^+^ and OH^−^ [10]. This proof of concept later led to the introduction of the first miniaturized silicon-based chemical sensor.

**Table 1:** Summary of field-effect-based DNA sensors discussed in literature.

Sensor Type	DNA Probe/Amplification Reaction	Target DNA	Ref. Electrode	Reference
***DNA-modified Field Effect Devices***				
*EIS; FET p-Si–SiO_2_*	Oligo(dT20); poly(dT1000bp)	Oligo(dA18); poly(dA) (1000 bp)	Ag/AgCl	[11]
*p-channel FET; n-Si–SiO_2_; silanization with APTES*	Adsorption ~4 × 10^11^ molecules/cm^2^	(20,45)-mer ssDNA	Ag/AgCl liquid-junction	[12]
*p-channel FET; n-Si–SiO_2_ poly-l-lysine*	20-mer dsDNA; 2 × 10^8^ molecules/cm^2^	dsDNA	Ag/AgCl wires	[13]
	10-mer ssDNA	20-mer ssDNA - mismatch detection	Liquid junction Ag/AgCl	[14]
*p-channel Au-gate (floating) FET; n-Si–SiO_2_*	(12,15)-mer thiol-modified ssDNA [2.8; 3.5] × 10^8^ molecules/cm^2^	ssDNA	Ag/AgCl	[15]
*p-Channel TFT / Poly-Si TFTs*	18-mer ssDNA probes [10^12^;10^13^] molecules/cm^2^	18-mer ssDNA - mismatch detection	Ag/AgCl	[16]
*a-Si:H ISFETs*	19-Mer ssDNA 6–60 × 10^12^ pmol/cm^2^	19-mer ssDNA - mismatch detection	Ag/AgCl micro ref. electrode	[17]
*n-channel depletion FET; p-Si–SiO2 –Si_3_N_4_; Au Gate*	Thiolated (15,25)-mer ssDNA	(15,25)-mer ssDNA	Ag/AgCl	[18,19]
	17-mer ssDNA/ 1.7 × 10^8^ molecules/cm^2^	17-mer ssDNA	Pt & Without RE	[20,21]
	20-mer dsDNA; 1.2 × 10^13^; Adsorption	400bp cDNA	Ag/AgCl	[22]
*Pentacene and poly(3-hexylthiophene) TFTs*	(20,21)-mer ssDNA	ssDNA	Without RE	[23,24]
*Flat device - CMFET; flexible substrates*	13-mer poly-dT spacer, 18-mer ssDNA	18bp ssDNA	Without RE (control gate)	[25,26,27]
*n-Type SiNW; p-type SiNW; SWNT*	ssDNA, PNA	RT-PCR product of DEN-2; microRNA; RCA (ssDNA)	-	[28,29,30,31,32,33,34,35,36,37,38,39]
*Graphene oxide FET*	PNA	LOD 100fM- mismatch detection		[40]
***Enzyme based Field Effect Devices***				
*ISFET- Si_3_N_4_*	SBE	dsDNA	Ag/AgCl	[41]
*CMOS- ISFET- Ta_2_O_5_ sensitive layer*	DNA Sequencing	dsDNA/cDNA	-	[6]
*CMOS- ISFET- Si_3_N_4_*; *EIS-Ta_2_O_5_*	Real-time qPCR	dsDNA/cDNA; cytochrome P450 SNPs; GH1; cMYC	Ag/AgCl	[42,43]
*CMOS- ISFET- Si_3_N_4_*; *EIS-Ta_2_O_5_*	Real-time qLAMP	dsDNA/cDNA; cytochrome P450 SNPs; NAT2; cMYC	Ag/AgCl	[43,44]

### 2.1. Operating Principle of Field Effect-Based Biosensors

FETs may be described as three electrode devices where the current flow between the source and drain electrodes can be modulated by varying the potential applied to the gate and source electrodes [9,10]. The semiconductor layer is separated from the gate electrode by an insulator layer that prevents current flow between them. The current-control mechanism is based on an electric field generated by the voltage applied to the gate [10]. The current is conducted by one type of carriers (electrons or holes) depending on the semiconductor’s type (n-type or p-type). In the case of a p-type semiconductor, when a positive gate voltage is applied electrons are repelled from the semiconductor- insulator interface creating a depletion layer that acts as an insulator, consequently negligible current flows between source and drain. However, applying a negative gate voltage attracts electrons to the semiconductor surface, when a sufficiently high concentration of electrons is accumulated in this region a conductive channel is created at the semiconductor-insulator interface allowing a current flow between source and drain. The opposite effect occurs in n-type semiconductors [9]. The gate voltage modulates the channel conductance and, as it is increased, the FET can be switched from what is conventionally called the OFF to the ON state. In the ON state the drain-to-source current varies with both the gate voltage and the drain voltage. Depending on the drain voltage value two regimes of operation can be distinguished: linear and saturation. The transfer characteristics, allow the determination of electrical parameters that characterize these devices performance (e.g., threshold voltage; carriers mobility; ON/OFF current ratio) [8]. In ISFETs, the most important parameter is the threshold voltage, as it is influenced by the flat-band voltage of the gate/semiconductor/insulator capacitor structure. In a non-ideal device the flat-band voltage is not usually zero and its value is affected by the existence of oxide charges; the work function difference between the semiconductor and the solution; the reference electrode potential; the surface-dipole potential of the solution and the electrolyte/insulator interface potential; which is dependent on the ion concentration in the solution [7].

A FED can be configured as a biosensor by modifying the gate terminal with molecular receptors or ion-selective layers for the analyte of interest. The binding of a charged biomolecule results in depletion or accumulation of carriers caused by change of electric charges on the gate terminal. The dependence of the channel conductance on gate voltage makes FEDs good candidates for electrical biosensors because the electric field generated from the biomolecule binding is analogous to applying a voltage to the gate [8]. Moreover, FEDs can be miniaturized without losing signal to noise ratio since the channel current of a FET is proportional to the width/length ratio of the channel and not related to the area of the device. Therefore, FEDs are ideal for the application in small-sized, high-density and multi-functional microarray sensors [23].

### 2.2. Oxide/Electrolyte Interface & Sensitive Layer

Independently from the used architecture one of the most common approaches in the application of field effect devices as biosensors is the replacement of the gate electrode by an electrolyte. The insulator, commonly an oxide, is thus directly exposed to the electrolyte so changes in the solution and/or at the interface can affect the oxide surface potential and modulate the device’s response. The relationship between the potential applied to an field effect sensor and the accumulated charge at the surface of electrochemical interfaces can be divided into two components; the electrostatic potential of ions in solution, as defined by the electrochemical double layer, and charge formation due to chemical reactions that occur at the oxide’s surface, as described by the site binding model [45].

In ISFETs, the oxide/electrolyte interface potential can modulate the threshold voltage. The difference in applied potential is distributed among various components: the electrolyte/oxide interface potential due to the electrochemical double layer (Gouy-Chapman-Stern model); the oxide surface potential (site-binding model); the potential drop thought the dielectric; the depletion charge potential drop in the semiconductor and the potential drops due to the electron affinities between the electrolyte and the semiconductor. Also, chemical processes can occur at the electrolyte/oxide interface affecting the semiconductor surface potential and thus modulate the devices response. The relation between this interface potential and the pH (hence the pH sensitivity) is mainly determined by the intrinsic buffer capacity of the oxide surface [3,8].

**Figure 2:**
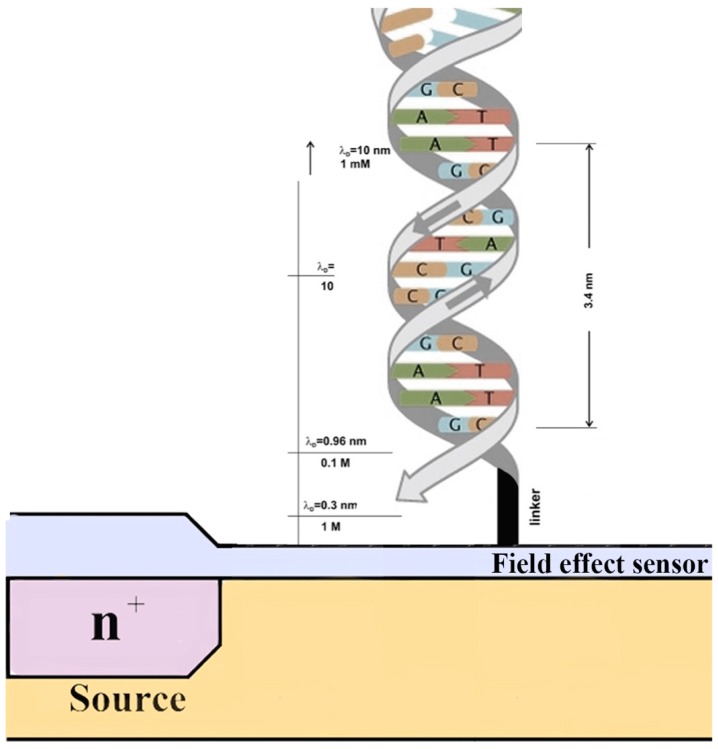
Schematic illustration of a DNA covalently bound to a sensor surface and Debye length (λ_D_) in electrolyte with different ionic strengths. Reduction of the buffer ionic strength allows for the detection of interaction events at larger distances from the sensor surface.

Under applied bias, charges accumulate on both sides of an electrode/electrolyte interface. The model for the potential distribution at the electrolyte side considers that the applied potential and the electrolyte concentration influence the double layer capacity [3,45]. The potential decreases exponentially with the distance from the electrode and the decay length. The also called Debye length can be described as the distance over which the electric field is screened by mobile charge carriers, such as electrons or ions in solution. At distances greater than this, charges in solution are balanced by counter-ions, resulting in a zero net charge. For biosensing purposes, this distance is very important because only events that occur between the sensor’s surface and the Debye length can be detected. In particular, the so called Debye shielding effect is closely related to the ionic strength of the buffer solution, since the surface charges of biomolecules in a buffer solution are shielded by oppositely charged buffer ions (Figure 2) [8].

The site binding model describes the component of the potential drop that arises from chemical processes at the surface of the insulator. Insulator materials that are used as sensitive layers for field-effect sensors are typically oxides (e.g., SiO_2_; Al_2_O_3_; Ta_2_O_5_). The surface of these oxides contains a large amount of active sites. These surface sites are amphoteric, meaning that they can act as proton donors or acceptors. Chemical processes that occur at the electrolyte/oxide interface give rise to a change in electrolyte/insulator surface potential, thus modulating the devices response. These chemical reactions occur within the outer Helmholtz plane (OHP) because chemical phenomena are limited to molecular distances. The only ionic species that can penetrate the OHP are hydrogen and hydroxyl ions, due to their small sizes and lack of solvation shell [8,46]. The relation between this interface potential and the pH, hence the pH sensitivity, is mainly determined by the intrinsic buffer capacity of the oxide surface.

### 2.3. Production Methods

The sensitivity of FEDs is modulated by the intrinsic characteristics of the sensitive layer material. Theoretically, any material with surface amphoteric groups such as oxides, can act as a sensitive layer for hydrogen ions in solution, hence as pH sensors. Historically the first materials studied as pH sensitive surfaces were commonly used field effect transistor gate dielectrics (e.g., SiO_2_, Si_3_N_4_, Al_2_O_3_ and Ta_2_O_5_) [10,47]. Several deposition methods, both chemical and physical, can be used to produce thin films of these materials. However, the production process and processing conditions influence the film’s properties, namely pH sensitivity and sensor performance [48].

**Figure 3:**
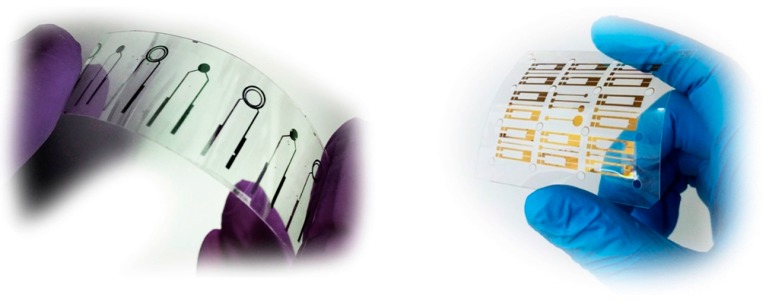
Flexible and disposable thin film field effect devices. Devices produced at CENIMAT/I3N, Universidade Nova de Lisboa by radio frequency magnetron sputtering in low cost flexible substrates according to the procedure described in [55,64].

Chemical based deposition methods include metal-organic chemical vapour deposition (MOCVD); atomic layer deposition (ALD) and solution based methods. From these The MOCVD and ALD offer several advantages such as good quality films with a controlled thickness. However, they also bring some disadvantages, since the precursors can be toxic and dangerous and high processing temperatures are usually needed [49,50,51,52]. Solution based methods, have the advantage of simplicity and do not require complex equipment. These methods are perfectly suited for low temperature fabrication, including conventional printing methods allowing integration with cheap and flexible substrates. However, reproducibility and high quality films are still difficult to obtain [53,54,55,56,57]. Physical methods such as thermal evaporation and sputtering represent an alternative for the production of ISFET devices. In fact, thermal evaporation of a metal layer followed by thermal oxidation can lead to good quality films but a high production temperature is needed [58,59]. Sputtering techniques such as radio frequency (rf) magnetron sputtering is perfectly suited for low temperature fabrication however dielectric materials are hard to sputter requiring high power density that might damage the growing film and interfaces. Nevertheless this deposition technique allows for quality films of a variety of materials to be obtained at room temperature being compatible with low-cost, flexible and disposable substrates [48,55,60,61,62,63,64] (Figure 3).

## 3. Applications of Field Effect Biosensors

Field effect devices are a promising technology for label free DNA analysis suitable for molecular screening of pathogens and diagnostics of genetic based disorders [15,65]. A wide diversity of field effect biosensors with variable architectures, sensitive layer composition, biological functionalization methods and biological target of detection have already been reported [10,66,67]. The change in DNA content, either due to hybridization or enzymatic reaction, yields a local pH/charge variation and a rearrangement of ionic species near the sensor surface that modulate the sensor’s response. Therefore, the most commonly used approaches for DNA analysis rely on enzyme-based FED and DNA-modified FED [68].

### 3.1. Applications of DNA-Modified Field Effect Devices

**Figure 4:**
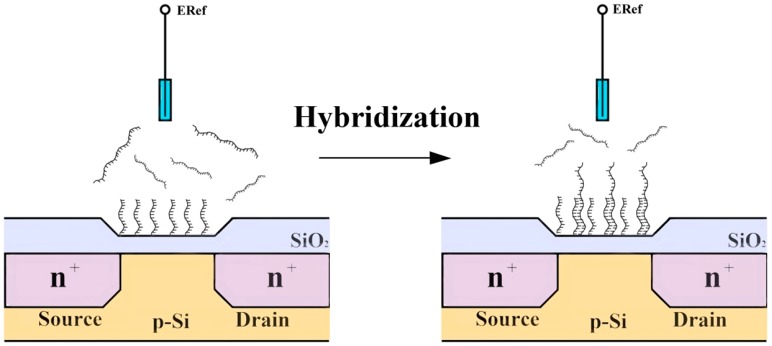
Schematic structure of a DNA modified thin film field effect device and the principle of DNA-hybridization detection. The change in DNA content, due to hybridization, yields a local charge variation and a rearrangement of ionic species near the sensor surface that modulate the sensor's response.

Most DNA detection techniques are based on a DNA hybridization process, where a specific single stranded DNA (ssDNA) molecules—probe—recognizes the complementary strand mediated by mostly Watson and Crick base pairing events. Detection of hybridization is usually achieved by DNA modified FED via immobilizing probes onto the sensor’s surface. Because DNA is an intrinsically charged molecule due to the phosphate backbone, the charge density increases near the sensor’s surface yielding a response (Figure 4) [3].

The DNA probe density on the sensitive area is one of the most relevant factors to take into account while developing such approaches. In principle, higher probe density ought to yield higher hybridized charge and enhanced sensor signal; however, if the density is too high, a decrease in hybridization efficiency occurs due to electrostatic repulsions [69]. Also, as previously discussed, the sensitivity in FET-type biosensors is strongly associated with the Debye screening length between the gate surface and the analyte solution [66]. The issue of the electric double layer shielding should be carefully considered in the design of FET-based biosensor applications, as biorecognition events, such as DNA hybridization, must occur within the Debye length.

#### 3.1.1. Thin Film Field Effect Devices

The development of DNA modified FED platforms for the label-free detection of covalent immobilization of DNA and subsequent hybridization to its complementary DNA have been widely reported [11,12,13,18,19,20,21,38,39,40,41,42,43,44,45,46,70]. Direct functionalization by covalent attachment of a self-assembled monolayer of DNA probes to the sensor surface (either gold or oxide based) is most commonly used [11,16,17,71,72]. Nonetheless, electrostatic immobilization of probe DNA has also been described [15,22,73,74].

Souteyrand and co-workers reported the first experimental results of DNA hybridization based on field-effect measurements [11]. In this specific approach, a thiol-modified ssDNA probe sequence was immobilized onto the Gold metal gate surface of a MOSFET device without any surface modification. Similar results were later reported by Kim and co-workers with a p-type MOSFET and by Sakata and coworkers with an n-type silicon FET with Si_3_N_4_ gate insulator [18,70]. In both cases, the drain current was significantly shifted after DNA immobilization and specific target hybridization. A similar concept used a MOS capacitors consisting of Au/SiO_2_/Si and Poly-Si TFTs with a gold metal gate as ISFET biosensor for label-free electrical detection of DNA hybridization [16]. Hybridization of target molecules to immobilized probes results in a change in potential of the interfacial dipole at the metal gate/electrolyte interface, thus modulating the device characteristics. Interestingly, by using this ISFET configuration with the appropriate DNA probes, the authors claim it is feasible to detect single-base DNA mismatches, opening the way for sensitive detection of single nucleotide polymorphisms (SNPs), one of the most frequent genetic alterations in the human population. Upon interaction of the ssDNA probe with a complementary strand containing a single base pair mismatch a statistically significant difference was observed, when compared to the fully complementary strand target (~60 mV) [16]. The capability of these devices to detect a single nucleotide mismatch has been since been reported for other device architectures and materials [14,24].

#### 3.1.2. New Materials and Designs

Recent studies have raised the issue of the limitations of classic charge detection of DNA hybridization based on field effect biosensors. In particular, parameters such as sensitivity, specificity, stability and production price led to the research of new materials and designs approaches [10,28,75].

Towards optimization of these platforms, nanostructured materials have been explored [28,75]. One such examples of wide application are nanowires (NW) that operate like typical FET-based sensors, only that the analyte affects the current flow through the whole of the diameter of the NW rather than just at the surface, as is the case with planar FETs, rendering them far more sensitive [28]. The main disadvantage of nanowire-based oligonucleotide sensors is that they are complex and expensive to fabricate [40]. However, nanowire-based FED devices are highly sensitive, promise fast response times and can be fabricated in arrays for multiplex detection into small volume platforms. Nanowire sensors are most commonly fabricated from silicon, but other materials have been gaining momentum, e.g., gold gallium nitride, graphene oxide and carbon nanotubes [29,30,31,32,33,34,35,36,37,38,75,76,77].

Hahm and Lieber developed the first p-type two-terminal silicon nanowire electronic device for specific and ultrasensitive DNA hybridization detection [33]. In this approach, the authors modified the surface of the silicon nanowire devices with peptide nucleic acid (PNA) receptors designed to recognize wild type *versus* the ΔF508 mutation site of the cystic fibrosis transmembrane receptor gene. Conductance measurements exhibited a time-dependent conductance change consistent with the PNA−DNA hybridization and enabled identification of fully complementary *versus* mismatched DNA samples. This approach shows that detection can be carried out at the femtomolar range allowing for the direct, label-free DNA detection with extreme sensitivity and specificity [33]. Also, Zhang and co-workers developed a highly sensitive and sequence-specific detection using nonoxidized silicon nanowires (SiNWs) and PNA probes. The purposed approach showed limit of detection down to 1 fM, with mismatched sequence discrimination capability allowing the detection of oligonucleotides of approximately 20 bases in length, using targets such as the miRNAs let7b and let7c (the deregulation of which is associated with various forms of cancer) and a gene fragment of the dengue virus [34,35,36,37,38]. Recently, Gao and co-workers used a similar approach achieving a Limit of detection (LOD) of 50 aM but this sensor requires the use of Rolling Circle Amplification, a rather complex reaction to setup, to selectively amplify a particular nucleic acid sequence to enhance the signal [39].

To overcome the limitation of the double layer shielding due to mobile ions present in the solution, Kulkarni and Zhong demonstrated a new high-frequency nanoelectronic sensing platform to overcome the ionic screening effect by operating a single walled carbon nanotube single-walled carbon nanotube (SWNT) field effect transistor as a high-frequency biosensor. This approach detects molecular dipoles at high frequency rather than the associated molecular charge. The nonlinear mixing between the alternating current excitation field and the molecular dipole field can generate mixing current sensitive to the surface-bound biomolecules. Moreover, the frequency mixing due to the nonlinear I−V characteristics of a nanotube FET allowed operating the sensor at frequencies high enough to overcome ionic screening and yet detect the frequency mixed signals at lower frequencies [23].

More recently, there has been growing interest in the use of organic thin film transistors (OTFT) for fabrication of low-cost FED biosensors. OTFTs are excellent candidates for use in disposable sensors since they are easy and cheap to fabricate when compared to their inorganic counterparts [71]. Organic materials can be dissolved in various solvents, so that transistors can be coated or printed at low temperature. In addition, organic semiconductors are biocompatible and flexible thus they can be integrated with biological systems [78].

Several types of OTFT-based DNA biosensors have been reported. Zhang and Subramanian reported on the first pentacene TFT DNA biosensor, in which DNA molecules are immobilized on the surface of semiconductor layer. This report shown the potential of organ thin film transistors for label-free DNA detection by showing different electrical performance shifts in response to single and double stranded DNA [74,78]. However, this approach may suffer from stability and repeatability issues since the pentacene film is sensitive to moisture and some ions. Recently several groups have followed this research line and achieved stable and sensitive devices [24,78,79,80]. Moreover, recent developments of the production process dramatically increased the sensitivity of pentacene-based DNA hybridization sensors, and coupled with a microfluidic system for an integrated genetic diagnostic tool [24,81].

Also, Cai and co-workers have developed a graphene-based gene FET that has demonstrated a limit of detection of 100 fM. For the first time a reduced graphene oxide based field effect transistor biosensor was coupled with peptide nucleic acid (PNA) probes for high sensitive and specific hybridisation. This sensor showed an increased sensitivity, allowing to improve the limit of detection in 1 order of magnitude than previous reports. Moreover, this device was able to detect single nucleotide mismatches and is capable of being regenerated [40]. Song and co-workers proposed a diamond solution-gated FET in which the DNA was immobilized directly onto amine-terminated sites. The diamond surface channel attached by DNA was exposed directly to the electrolyte lacking gate insulator. The tested device could rapidly detect 3-mer mismatched DNA, and showed the possibility of single-base mismatched DNA detection, without losing sensitivity [82].

New design approaches have introduced some major improvements to FED-based detection, namely, the introduction of active FEDs that can interact with the sample changed the mind-set behind the design principle. This new concept is based on a simple premise that the sensor can actively interact with the analyte to improve speed, sensitivity and specificity of the biological reaction. Fixe and co-workers proposed the use of pulse-enhanced DNA hybridization to dramatically speed up the analysis process [25,76]. This new strategy allowed for the detection of on-chip single-base mismatches in hybridized duplexes, using a single square voltage pulse that simultaneously increases the rate and specificity of the hybridization process [24,25].

Detection of the intrinsic molecular charge of analytes has been proposed for field-effect measurements. The main drawback of the majority of the design implementations is the need for reference counter-electrodes, which cannot be easily integrated in a standard CMOS process. This issue prevents the effective application in low-cost, disposable devices. For example, Barbaro and co-worker developed a solid state device able to detect changes of electric charge with no need for any external component such as a reference electrode [26]. The proposed (charge-modulated field-effect transistor ‒ CMFET) is based on an evolution of the floating-gate transistor coupled with a control-gate with the role of reference electrode and an active area activated by charge induction (Figure 1C). The entire device can be produced by standard CMOS technology providing low-cost, large-scale-of-integration capabilities. This concept was used for the direct electronic detection of DNA hybridization, showing high sensitivity to any charge variation occurring in the vicinity of the DNA layer, including a hybridization event [27,83].

### 3.2. Applications of Enzyme Based Field Effect Devices

The concept of combining the specific biocatalytic capabilities of enzymes with field effect devices was first proposed by Janata and Moss in 1976 [84]. Since then, a large variety of enzymes that allow the detection of numerous analytes (e.g., glucose, penicillin, urea, pesticides, *etc.*) have been used in numerous platforms [4,7,10,66,67,85,86].

Most current nucleic acid diagnostic methods rely on the possibility of DNA amplification via the polymerase chain reaction (PCR), which has since been considered the gold standard in nucleic acid detection [82,83]. The development of Real-time PCR has allowed for target DNA template quantification in real-time through fluorescence [42,87,88,89,90].

**Figure 5:**
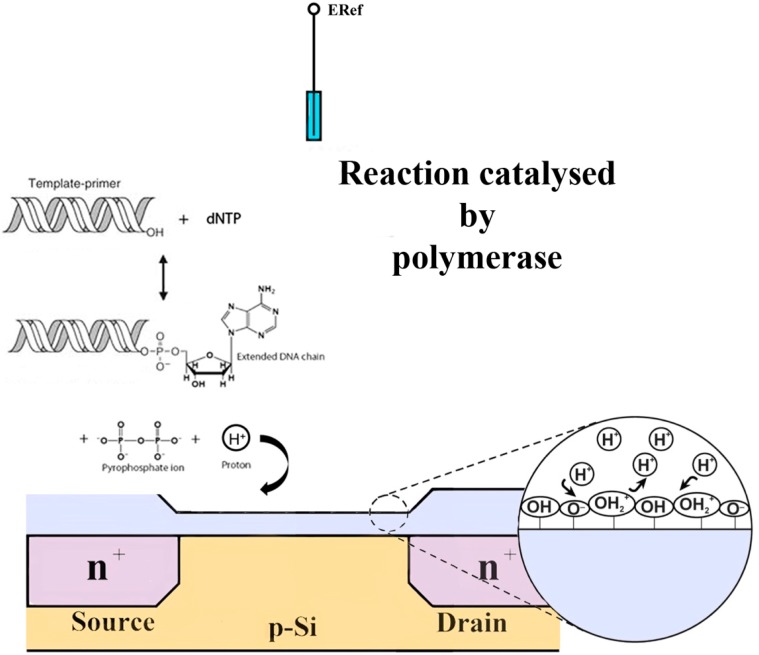
Structure and principle of function of an enzyme-based thin film field effect device. The change in DNA content, due to an enzymatic reaction, yields a local pH variation and a rearrangement of ionic species near the sensor surface that modulate the sensor’s response.

The use of FED sensors coupled to PCR for real-time detection, quantification and characterization of DNA without the need for additional labeling and/or reporter molecules has become an exciting approach. The detection mechanism is based on the fact that the incorporation of a nucleotide into a strand of DNA by a polymerase releases a hydrogen ion as a by- product; resulting in a local pH variation that can be detected by the underlying ion sensitive FED and converted to a measurable signal (Figure 5). Examples include DNA sequencing equipment—IonTorrent^TM^ [5,6], real-time monitoring of PCR amplification [43,44] and isothermal DNA amplification [41,44].

Purushothaman and co-workers reported on a new method for identifying SNPs using ISFET technology. The key innovation was the integration of a single base extension reaction with a commercial ISFET (Sentron Europe BV) to detect the incorporation of a single nucleotide, which is compared to the output from a non-expected base incorporation signal [91]. A single ISFET with a silver/silver chloride reference electrode was used and the pH change was measured in a very weakly buffered reaction. More recently ISFET devices have been applied in the label-free DNA sequencing of genomes and subsequent application of this technology to a commercially available DNA sequencing equipment—IonTorrent^TM^ [5,6]. The IonTorrent uses a high-density array of wells, each holding a different DNA template, to perform this biochemical process in a massively parallel way; beneath the wells lies an array of ISFETs fabricated by standard CMOS technology. The ISFET directly detects the sequencing event thus eliminating the need for fluorescent labels and complex optical detection apparatus.

Despite the increasing use of electrochemical DNA detection approaches, only a few have been directed towards gene expression analysis [92,93,94]. Very recently, two groups reported on the use of FEDs for real-time amplification and detection of nucleic acid using pH sensing. Toumazou and co-workers developed an integrated chip for real-time amplification and detection of nucleic acid using pH sensing complementary metal-oxide semiconductor (CMOS) technology [44]. The developed chip includes ion-sensitive field effect transistor sensors, temperature sensors, resistive heating, signal processing and control circuitry all integrated to create a full system-on-chip platform. The platform was evaluated using two amplification strategies (PCR and loop mediated isothermal DNA amplification–LAMP) to genotype and discriminated unique single-nucleotide polymorphism variants of the cytochrome P450 family from crude human saliva [44]. Veigas *et al*. reported on the development of a Ta_2_O_5_ EIS sensor for label free real-time quantitative LAMP DNA amplification towards gene expression profiling, without the need for additional labeling and/or reporter molecules. This work demonstrated the potential to quantify in real-time cMYC, a proto-oncogene amplified and overexpressed in most human cancers. Successful LAMP amplification of cMYC was achieved in a specifically developed isothermal amplification cell with an in-house temperature control setup. The amplification reaction was monitored in real-time with the optimized Ta_2_O_5_-based sensor and a clear discrimination of template DNA initial concentration was observed [41].

## 4. Future Perspectives

Several sensor field effect detection systems with different configurations and architectures have been proposed to be applied in the selective and specific detection of nucleic acids. Such systems show the promise to revolutionize DNA/RNA based diagnostics. Such developments suggest the possibility of cheap production processes and material to further reduce the overall cost of molecular diagnostics. The use of low temperature fabrication processes will pave the way to produce FEDs on flexible low cost substrates, such as biodegradable polymers, which show excellent properties [56,64,95,96,97]. The remarkable development of organic and oxide semiconductors based devices and their application in biosensing may established these as a new generation of electronic devices, providing an attractive alternative for DNA based biosensors [95,96,97].

The development of new biomolecular probes and enzymatic amplification procedures may lead to the development of new strategies. In particular, the use of isothermal DNA amplification techniques may lead to a new generation of devices with low energy requirements, while achieving LODs compatible with molecular diagnostics at peripheral labs. Optimization and integration of this amplification approaches into a suitable platform could significantly lower the costs associated with gene expression analysis and consequently allowing for the molecular diagnostics of cancer at point-of-need.

FEDs-based biosensors have become one of the central technologies for DNA detection and characterization. However, these devices still need to be improved for better performance with regard to real sample detection and multiple-sample detection. The step-by-step improvement on the fabrication of these devices and continuous optimization of operation parameter led to the elimination of nonspecific molecular interaction, contributing to an improvement in the sensitivity, specificity and reliability of this class of biosensors. The introduction of new concepts in design procedures and materials introduced new working prototypes that may lead the future of DNA biosensing. The integration of full solid-state devices without the need for traditional reference electrodes with new materials compatible, low temperature and low cost fabrication methods (e.g., low cost printing technologies) and low temperature isothermal DNA/RNA amplification techniques will soon introduce a new class of cheap handheld DNA micro-array devices.
